# Self-Assembly of Silver Clusters into One- and Two-Dimensional Structures and Highly Selective Methanol Sensing

**DOI:** 10.34133/research.0018

**Published:** 2022-12-21

**Authors:** Zhaoxian Qin, Zhiwen Li, Sachil Sharma, Yongwu Peng, Rongchao Jin, Gao Li

**Affiliations:** ^1^ State Key Laboratory of Catalysis, Dalian Institute of Chemical Physics, Chinese Academy of Sciences, Dalian 116023, China.; ^2^ Department of Chemistry, Carnegie Mellon University, Pittsburgh, PA 15213, USA.; ^3^ College of Materials Science and Engineering and College of Chemical Engineering, Zhejiang University of Technology, Hangzhou 310014, China.; ^4^ University of Chinese Academy of Sciences, 100049 Beijing, China.

## Abstract

The development of new materials for the design of sensitive and responsive sensors has become a crucial research direction. Here, two silver cluster-based polymers (Ag-CBPs), including one-dimensional {[Ag_22_(L1)_8_(CF_3_CO_2_)_14_](CH_3_OH)_2_}*_n_* chain and two-dimensional {[Ag_12_(L2)_2_(CO_2_CF_3_)_14_(H_2_O)_4_(AgCO_2_CF_3_)_4_](HNEt_3_)_2_}*_n_* film, are designed and used to simulate the human nose, an elegant sensor to smells, to distinguish organic solvents. We study the relationship between the atomic structures of Ag-CBPs determined by x-ray diffraction and the electrical properties in the presence of organic solvents (e.g., methanol and ethanol). The ligands, cations, and the ligated solvent molecules not only play an important role in the self-assembly process of Ag-CBP materials but also determine their physiochemical properties such as the sensing functionality.

## Introduction

New functional materials are of great importance for the design of highly sensitive and high-precision sensors for chemicals. The selective, precise, and swift measurement of volatile alcohols is critical in various areas such as the food industry [1,2], occupational safety [3], and forensics [4,5]. Toward this direction, the multidimensional nanoscale materials have garnered a lot of attention owing to their peculiarity in structures and properties, such as atomic/molecular thickness, optical transparency, and large surface areas [6,7]. The fabrication of alcohol sensing devices is an important aspect for the application of these materials. For instance, graphene-based nanomaterials are considered as one of the frontiers of exploring the sensing materials [8–11]. The change in conductivity of graphene-based materials upon the adsorption of gas molecules leads to the gas detection. Besides this, some other 2-dimensional (2D) materials including nanostructures based on metal oxides [12,13], nanoporous silicon [14], hybrid carbon-based nanostructures [15], metal organic frameworks [16], and hybrids of fiber optics with nanostructures [17] have also displayed great promise in sensing of alcohols.

Recently, the burgeoning metal nanoclusters with well-resolved crystal structures show great prospects in fundamental research and applications in various aspects including luminescence, medicine, catalysis, energy, and biology [18–24] owing to their unique physiochemical properties. Meanwhile, multidimensional (e.g., 1D, [26–29], 2D, [27,30–34], and 3D [30,35–42]) self-assembled structures of metal nanoclusters have recently been designed to tailor their properties. For example, Mak and co-workers [30–32] have reported much work on the synthesis and structural determination of multidimensional silver nanocluster-based materials. Sun and co-workers [36,37] reported a series of multidimensional self-assembled nanocluster-based clusters with polyoxometallate as an anionic template, where organic ligands and halide atoms were used as linkers to connect clusters. Zhu and co-workers synthesized two 3D structures composed of [Au_1_Ag_22_(SAdm)_12_](SbF_6_)_2_Cl and [Au_1_Ag_22_(SAdm)_12_](SbF_6_)_3_ (SAdm = adamantanethiolate) as structural units [38]. These metal cluster-based materials have been well-developed as fluorescence probes to detect organic compounds [23–24] due to their unique fluorescence properties. However, the study of metal cluster-based materials applied as electrochemical sensors is still quite rare so far.

Here, we present the synthesis of 2 novel silver cluster-based polymers (Ag-CBPs) with atomically precise structures, including {Ag_22_(L1)_8_(CF_3_CO_2_)_14_(CH_3_OH)_2_}*_n_* (L1 = 3-(prop-2-yn-1-yloxy)-benzonitrile) chains and {[Ag_12_(L2)_2_(CO_2_CF_3_)_14_(H_2_O)_4_(AgCO_2_CF_3_)_4_]-(HNEt_3_)_2_}*_n_* (L2 = 1-(3-mercaptoprop-1-en-2-yl)-2-methoxypyridin-1-ium) film (abbreviated as Ag_22_-CBP and Ag_16_-CBP, respectively), which are determined by single-crystal x-ray diffraction. Interestingly, they show different conductivity under an external voltage in the presence of different organic solvents, which is related to their structural differences. We further find that the charge transfer and the species of charge carriers have an important influence on the conductivity of Ag-CBPs. The different responses of Ag-CBPs to the variation of organic solvents hold promise in the design of sensitive sensors for distinguishing solvents like the nose to differentiate smells.

## Materials

All chemicals were commercially available and were used as received without further purification. 3-Hydroxybenzonitrile (99%), 2-hydroxybenzonitrile (99%), silver trifluoroacetate (AgCO_2_CF_3_, 99%), silver tetrafluoroborate (AgBF_6_, 99%), 1-(3-mercaptoprop-1-en-2-yl)-2-methoxypyridin-1-ium bromide (99%), methanol (HPLC grade, 99.9%), ethanol (HPLC grade, 99.9%), acetone (HPLC grade, 99.9%), and toluene (HPLC grade, 99.9%) were purchased from Adamas-beta. Hydroxypropyl methylcellulose (HPMC) was received from Aladdin. Polyethylene terephthalate (PET; thickness of 125 μm) was obtained from Kangde Xin Composite Material Group. Ultrapure water (resistance, 18.2 MΩ·cm) was purified with a Barnstead Nanopure Di-water TM system.

## Synthesis of Ag_22_-CBP and Ag_16_-CBP

All the operations were carried out in dark. In general, 10 mg of Ag-L1 was dispersed into 5 ml of MeOH, followed by the addition of AgCO_2_CF_3_ (88 mg, 0.4 mmol, dissolved in 5 ml of MeOH). A white suspension was generated. After 20 min later, filtration of the mixture gave a light-yellow solution, which was then exposed to ethyl ether for crystallization in the dark in a refrigerator. Transparent yellow block-like crystals of {Ag_22_(L1)_8_(CO_2_CF_3_)_14_(CH_3_OH)_2_}*_n_* (denoted as Ag22-CBP) were obtained in a few weeks. {[Ag_12_(L2)_2_(CO_2_CF_3_)_14_(H_2_O)_4_(AgCO_2_CF_3_)_4_](HNEt_3_)_2_}*_n_* (Ag16-CBP) was obtained by a similar procedure. Briefly, 10 mg of Ag-L2 (dispersed in 5 ml of MeOH) was used in the preparation of Ag16-CBP. Light-yellow plate-like crystals of Ag_16_-CBP were obtained after weeks in the dark in a refrigerator. The synthetic yields of Ag_22_-CBP and Ag_16_ were 41% and 62% (based on consumption of AgCO_2_CF_3_), respectively.

## Sensor fabrication

For the preparation of the cluster thin-film sensor, 30 mg of the clusters was first dispersed in 5 ml of ethanol, and then 5 ml of HPMC aqueous solution (4 mg·ml^−1^) was added to adjust the viscosity. Next, 3 ml of cluster dispersion obtained from the previous step was dripped on PET and dried at 60 °C in an oven for 10 min. Finally, Cu wires were attached to the two ends of the film for connecting to the power supply in electrical measurements. The film of Ag-CBP sensors was sprayed into solvents. The pure solvents of methanol, ethanol, acetone, and toluene in HPLC grade and ultrapure water (resistance, 18.2 MΩ·cm) were used for the dynamic response and recovery of Ag-CBP sensors. The response time was 8 s, and the recovery time was 14 s. The sensitivity could be calculated as relative capacitance change for , where δ is relative current change *I*_x_ and *I*_0_ are measured currents when the sensor contacted with solvents and the initial current of free sensor (before dipping into solvents), respectively.The conductivity of the samples in solution was determined by a DDS-307 conductivity meter. Crystal samples were dispersed into different solution and then filtrated with a pinhole membrane filter, generating saturated solutions to be tested. Before the measurement, the conductivity meter was calibrated by a standard solution of 1408 μS·cm^−1^.

## Results and Discussion

### Atomic structure of Ag-CBPs

The cluster-based polymeric materials in this study with compositions of Ag_22_-CBP and Ag_16_-CBP were synthesized through a bottom-up synthetic strategy. Briefly, these polymeric materials were produced by the reaction and self-assembly of the corresponding silver precursors (Ag-L1 and Ag-L2) with AgCO_2_CF_3_, respectively (see the Supplementary Materials for details). Here, the alkynylate and thiolate ligands are selected to construct highly stable Ag-CBPs for their strong interaction and the flexible coordination between ligands and Ag atoms [31].

**Fig. 1.**
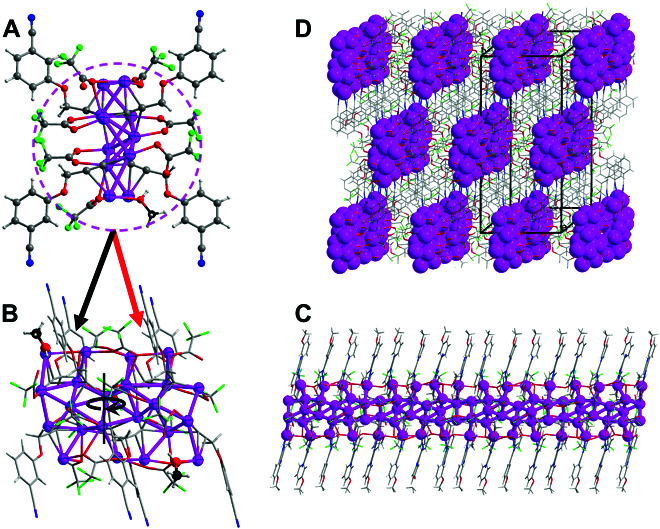
Structural anatomy of Ag_22_-CBP. (A) The asymmetric structure of Ag_22_-CBP. (B) The monomer structure of Ag_22_-CBP. (C) 1D silver chain along the *c* axis. (D) 3D structure with holes. Color code: Ag, purple; F, cyan; O, red; N, blue; C, gray and black; H, white.

The compositions and atomic structures of the Ag-CBPs are determined by single-crystal x-ray diffraction. The Ag_22_-CBP crystallizes in the *C*2*/c* space group. The minimum asymmetry unit of Ag_22_-CBP is constructed by 4 components, including 11 Ag atoms, 7 trifluoroacetate anions, 4 L1 anions, and a methanol molecule, as depicted in Fig. 1A. An asymmetry unit rotates by 180° around a *C*_2_ axis forming the monomer of Ag_22_-CBP (Fig. 1B). Thus, a metal framework of 22 Ag atoms is furnished in the monomer, where the distances between the silver atoms range from 2.798(4) Å to 3.353(3) Å. The monomers connect with each other in a head to tail manner, giving rise to a 1D silver chain along the ***c*** axis (Fig. 1C) through Ag–Ag bonds [3.302(4) Å], Ag–O–C(CF_3_)–O–Ag and Ag–O_trifluoroacetate_–Ag motifs, and Ag–alkynylate bonds (Fig. S1), which resembles a millipede. The obtained silver chains are covered by L1 ligands and trifluoroacetate ions through various bonds. For the L1 ligands, both the terminal C≡C and C≡N groups are bonded to Ag atoms (Fig. S2). Three types of coordination modes of terminal C≡C group are observed: (a) the *μ*_4_-*η*^1^, *η*^1^, *η*^2^, *η*^2^ mode (Fig. S3A), in which the C≡C group bonds to 2 Ag atoms via *σ* bonds and to another 2 Ag atoms through *π* bonds; (b) the *μ*_5_-*η*^1^, *η*^1^, *η*^1^ *η*^2^, *η*^2^ mode (Fig. S3B), in which the C≡C group links to 3 Ag atoms via *σ* bonds and to another 2 Ag atoms through *π* bonds; and (c) the *μ*_4_-*η*^1^, *η*^1^, *η*^1^, *η*^2^ mode (Fig. S3C), in which the C≡C group is bonded to 3 Ag atoms via *σ* bonds and to another Ag atom through *π* bond. Only the C≡N–Ag *σ* bonding mode is detected between the C≡N group and Ag (Fig. S2). Besides, 3 coordination modes are observed between trifluoroacetate ions and Ag atoms, namely, *μ*_1_–O_trifluoroacetate_, *μ*_2_–O_trifluoroacetate_, and *μ*_3_–O_trifluoroacetate_ bonding modes. Besides L1 ligands and trifluoroacetate ions, it is worth noting that the nonionized methanol molecules bind with Ag atoms through Ag–O_methanol_ bonds [2.425(2) Å], which are shorter than the Ag–O_trifluoroacetate_ bonds [2.688(5) Å] (Fig. 1A and Fig. S4). These obtained silver chains are discrete but bridged to each other by C≡C groups and C≡N groups of L1 ligands to form the final 3D structure of Ag_22_-CBP, as shown in Fig. 1D.

**Fig. 2.**
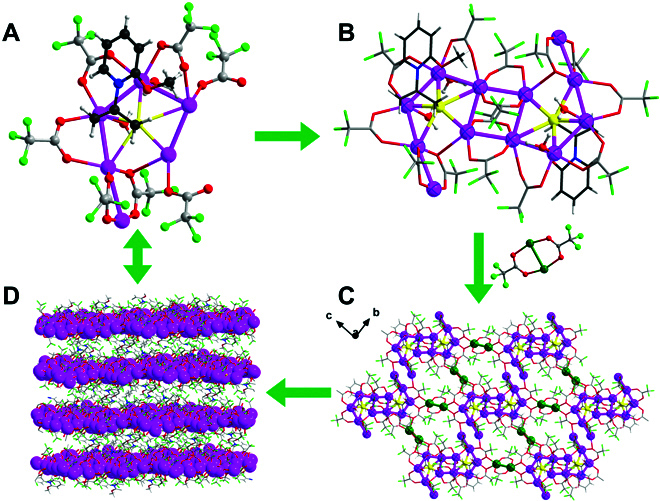
Structural anatomy of Ag_16_-CBP. (A) Ag_6_ unit in asymmetry. (B) Ag_12_ unit in monomer. 2D structure (C) and 3D structure (D) of Ag_16_-CBP. Color code: Ag, purple and green; S, yellow; F, cyan; O, red; N, blue; C, gray and black; H, white.

A 2D structure of Ag_16_-CBP in *P-*1 space group was obtained when the bidentate L1 ligand was replaced by the unidentate L2 ligand under the otherwise similar experimental conditions. As shown in Fig. S5, 8 Ag atoms, 9 CF_3_CO_2_^−^ ions, 1 L2 ligand, 2 aqua molecules and 1 [HNEt_3_]^+^ are found in the minimum asymmetry unit. The S atom of L2 ligand bonds to 5 Ag atoms via Ag–S bonds [2.447(3) to 2.979(2) Å], forming the main body of an inverted Ag_6_ unit as depicted in Fig. 2A and Fig. S6A, where the Ag–Ag bond length is in the range of 2.936(4) to 3.043(3) Å. Furthermore, 2 Ag_6_ units connect with each other, generating a *Z*-motif Ag_12_ cluster (Fig. 2B and Fig. S7), which is covered by 14 trifluoroacetate ions via Ag–O bonds [2.215(3) to 2.428(5) Å]. A molecular cavity is formed, where 2 water molecules and 1 [HNEt_3_]^+^ ion settle down through Ag–O_water_ bonds of 2.481(5) to 2.669(3) Å and hydrogen bond with a D---A distance of 2.798(3) Å between the [HNEt_3_]^+^ ion and aquo molecule (Fig. S8). These Ag_12_ clusters relate to each other by head to tail along the ***b*** axis via (Ag_12_)–O_triflouroacetate_–(Ag_12_) motifs (Fig. 2C), where the shortest Ag---Ag distance between Ag_12_ clusters is ~3.572 Å, beyond the limit of Ag–Ag bonds. Furthermore, the Ag_2_(CF_3_CO_2_)_2_ units (noted as Ag_2_, highlighted in green in Fig. 2 and Fig. S6B) serve as the linkers to connect neighboring Ag_12_ clusters in the ***bc*** plane, i.e., the Ag_12_ clusters interact with 4 neighboring Ag_12_ clusters through 4 Ag_2_ units by the (Ag_12_)–O_triflouroacetate_–(Ag_2_) motifs (Fig. 2c) to form a 2D plate. Finally, the 2D plates are packed into the 3D structure through hydrogen bonds and Van der Waals forces between layers (Fig. 2D).

### Sensing response to alcohol

Different from the common silver complexes [42], silver clusters [18,19] and even silver wires [22], here, we have obtained the silver frameworks, silver chains in Ag_22_-CBP, and silver plates in Ag_16_-CBP with unique and continuous features, which provide good channels for electrons to go through and prompt us to study their conductivity in the solid state.

To further confirm the structure uniformity and purity of the obtained Ag_22_-CBp and Ag_16_-CBP samples, we performed powder x-ray diffraction (PXRD) of Ag_22_-CBP and Ag_16_-CBP with their crystals and compared with simulated ones from the single-crystal structures as shown in Figs. S9 and S10. For both Ag_22_-CBP and Ag_16_-CBP, their experimental PXRD profiles match well with the simulated curves, respectively, indicating the high purity of crystal phases and structural consistency in Ag_22_-CBP and Ag_16_-CBP. Besides, we recorded and compared the PXRD patterns of the bulk crystal and powder samples after grinding. The data showed that the structures of Ag-CBPs were the same before and after the grinding (vide infra).

**Fig. 3.**
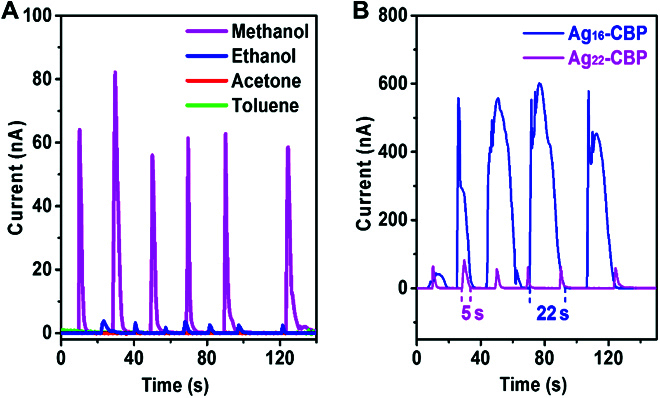
Dynamic response and recovery characterization of Ag-CBP sensors. (A) Dynamic response and recovery curves of Ag_22_-CBP thin-film sensor under different organic solvents (protic solvents: methanol and ethanol; aprotic solvents: acetone and toluene). (B) Dynamic response and recovery curves of Ag_22_-CBP and Ag_16_-CBP films in the presence of methanol. All the sprays were measured at 0.1-V bias.

We investigated the sensing property of the 1D Ag_22_-CBP chains and 2D Ag_16_-CBP plates on a home-made setup in a clean room at the ambient temperature of ~24 °C. During the tests, the powder of Ag-CBPs was painted on a flexible Polyethylene terephthalate board, which was further connected to a SourceMeter 2450 through Cu wires linking to polymer film on PET board, forming a closed circle (Figs. S11 and S12). Initially, the films made of Ag_22_-CBP and Ag_16_-CBP were insulating under dry conditions (as the reference). Once organic solvents (including protic solvents: methanol and ethanol; and aprotic solvents: acetone and toluene) were sprayed on the films, the film conductivity changes, and the film becomes conducting for methanol and ethanol but still insulating for acetone, toluene, and ultrapure water (resistance of 18.2 MΩ·cm) (Fig. 3 and Fig. S13). As shown in Fig. 3A, the electric current turns to zero again with the removal of methanol or ethanol from the thin films, indicating that the protic organic solvents should interact with Ag_22_-CBP and Ag_16_-CBP to make them conductive. On the contrary, the current was almost zero when acetone, toluene, and ultrapure water were sprayed on the films (Fig. 3A and Fig. S13), which corroborates that the Ag_22_-CBP and Ag_16_-CBP cannot detect these solvents, as these solvents cannot make the Ag_22_-CBP and Ag_16_-CBP nanomaterials conductive. The limit of detection is also evaluated in the methanol/water mixtures with different ratios, as shown in Fig. S13. The relative capacitance change of the Ag_16_-CBP sensor for methanol can reach up to 5,000 for the mixture of methanol and water (*v*A_methanol_/*v*_water_ = 3:7), which is ~33-fold higher than the reported cellulose/graphene nanocomposite [43].

The different responses to organic solvents suggest that both Ag_22_-CBP and Ag_16_-CBP could serve as good sensors for the protic organic solvents (e.g., methanol and ethanol), like the nose to recognize different smells in air. In comparison, Ag_22_-CBP and Ag_16_-CBP nanomaterials showed excellent dynamic responses to the methanol and ethanol detection, which may be due to the robust nature of these Ag-CBP nanomaterials. It is worthy to note that the responding electric current in methanol sensing is 15- to 25-fold that of the ethanol detection, and the signal falling time for methanol is longer as well (Figs. S14 and S15), which may be related to the stronger interaction between methanol and Ag_22_-CBP or Ag_16_-CBP than that of ethanol. The small cavities in Ag_22_-CBP (2.93 Å × 4.95 Å) and Ag_16_-CBP (3 Å × 3 Å) formed by the surface ligands of silver frameworks are much more suitable for small molecules like methanol and water only. In contrast, larger molecules such as ethanol require larger cavities than 3 Å.

Furthermore, the electric current intensity of the Ag_16_-CBP film (about 600 nA) is ~10-fold that of the Ag_22_-CBP film (~60 nA) with almost the same size under the identical experimental conditions, and the recovery time of Ag_16_-CBP film (~22 s) is much longer than that of Ag_22_-CBP film (~5 s) (Fig. 3B). All these results suggest that the Ag_16_-CBP film is much more sensitive to methanol than the Ag_22_-CBP film, which should be caused by the methanol absorption capability of the sensing material, for which the factors including the higher density of Ag atoms in the structural arrangement, richer hydrogen bonds, ion features, and the mass transfer in the 2D film of the Ag_16_-CBP should also be responsible.

**Fig. 4.**
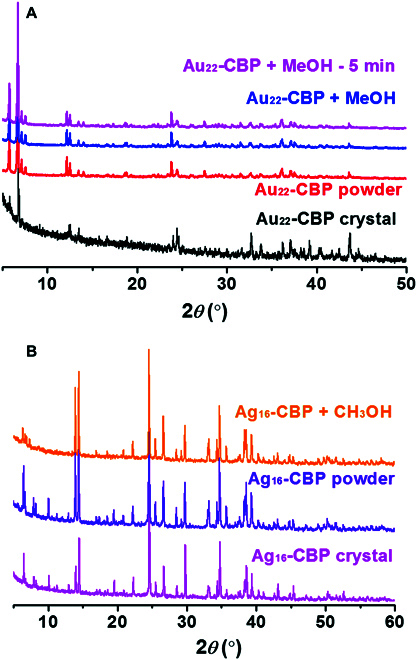
(A) PXRD of Ag_22_-CBP tested before and after the spray of methanol. (B) PXRD of Ag_16_-CBP tested before and after the spray of methanol.

To reveal how the conductivity of the CBP was affected by methanol, we studied the structural variation of CBP films with PXRD as shown in Fig. 4. Powders of Ag_22_-CBP and Ag_16_-CBP were made into films on a sample stage and tested. For Ag_22_-CBP, the powder sample presented more obvious signals at low angles than the bulk crystal samples, which is related to the size of sample and crystal face exposed. Ag_22_-CBP samples show the same profiles in bulk crystal and powder status, indicating that Ag_22_-CBP shares the same structures in powder and crystal. After methanol spray, a subtle decrement in the degree of crystallinity was detected, suggesting that partial crystalline Ag_22_-CBP maybe turns into amorphous in the presence of methanol although the structure of Ag_22_-CBP keeps inert. Besides, no further loss in the degree of crystallinity of Ag_22_-CBP film occurred even using more methanol, and the loss is irreversible. The situation for the 2D Ag_16_-CBP is more complicated. Some peaks in the 5° to 20° range in PXRD formed and disappeared besides the loss of degree of crystallinity. X-ray diffraction peaks at 6.6°, 7.94°, 8.2°, 10°, and 19.5° disappeared after methanol spray, and 4 new peaks were found at 6.4° to 7.5° simultaneously. These results indicate that the size/structure of Ag_16_-CBP has been affected by the sprayed methanol, which may be related to the 2D structure of Ag_16_-CBP. For example, the distance between layers and the relative position of layers may be affected by inducing methanol molecular.

### Mechanism

**Fig. 5.**
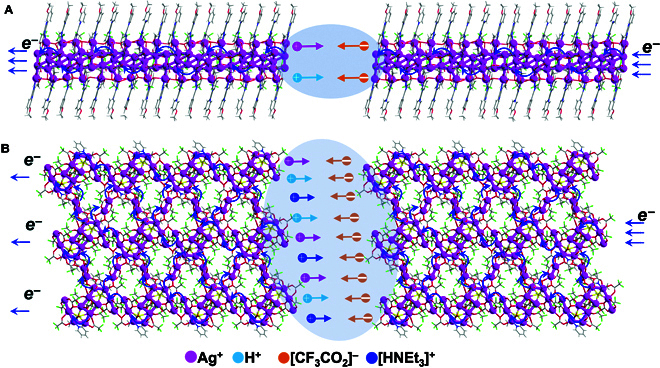
Mechanism of the charge transfer in the Ag-CBPs. The pathway for charge transfer in 1D silver (A) of Ag_22_-CBP and 2D Ag_16_-CBP (B). Notes: Ag^+^, purple ball; H, light blue ball; [CF_3_CO_2_]^−^, orange ball; [HNEt_3_]^+^, blue ball; microelectrolytic tanks, deep colored areas.

The sensitivity of sensors to solvents is closely related to the conductivity of Ag-CBP materials with solvents. Compared with the silver chains (1D) (Fig. 5A) in Ag_22_-CBP, the silver planes (2D) (Fig. 5B) in Ag_16_-CBP are more convenient for electrons to transfer in solid state under the applied voltage, generating a current. Furthermore, the positive pyridine rings and [HNEt_3_]^+^ in Ag_16_-CBP are helpful for the transfer of carrier, beside the silver planes. In fact, the dry Ag-CBP powders are insulators since the silver chains in Ag_22_-CBP and the silver planes in Ag_16_-CBP are disconnected, like a mess of broken electric wires, and no current can be generated even when a voltage is applied. However, once the Ag-CBP powders become wet by some solvents (such as methanol and ethanol would bond with the Ag atoms via Ag–O interactions), numerous microelectrolytic tanks (deep colored areas in Fig. 5) are formed among the broken silver chains and planes in Ag-CBPs, where ions dissociate from the Ag-CBPs and work as carriers in the electrolyte solution. Therefore, the solvent sensitivity of sensors made of Ag-CBPs depends on the conductivity of microelectrolytic tanks among silver chains and planes in Ag-CBP nanomaterials, as the charge transfer in solids of Ag-CBPs is immune from solvents outside.

The saturated methanol solutions of Ag_22_-CBP and Ag_16_-CBP were used to simulate microelectrolytic tanks between silver chains (1D) in Ag_22_-CBP and the silver planes (2D) in Ag_16_-CBP, respectively. The conductivity of saturated methanol solution of Ag_16_-CBP was determined as 608 μS·cm^−1^, ~3-fold that of Ag_22_-CBP (228 μS·cm^−1^), indicating that the microelectrolytic tanks among silver planes in Ag_16_-CBP showed better conductivity than that among silver chains in Ag_22_-CBP. For the better conductivity of Ag_16_-CBP in solution, the following structural factors may be responsible: (a) The species of carriers (Ag^+^, CF_3_CO_2_^−^, [HNEt_3_]^+^, and even H^+^ or [H_3_O]^+^ in Ag_16_-CBP) could serve as carriers in solution, but only Ag^+^ and CF_3_CO_2_^−^ could be disassociated from Ag_22_-CBP and serve as carriers in solution. (b) In the coordination modes of Ag, all the Ag^+^ cations were linked to L1 ligands through strong σ and π bonds, which is hard for Ag^+^ to disassociate, while the Ag_2_(CF_3_CO_2_)_2_ units are free to generate ions in Ag_16_-CBP except the Ag^+^ ions being bonded to L2 ligand via Ag–S bond. (c) The ligated waters and [HNEt_3_]^+^ ions in Ag_16_-CBP could provide more H^+^ ions, which are the fastest ionic carrier (in terms of mass transfer) in solution. All these structural factors determine the superior conductivity of Ag_16_-CBP in solution and more sensitive response to methanol. Furthermore, the conductivity of Ag-CBPs in ethanol was measured as ~50 μS·cm^−1^, ~0.45 μS·cm^−1^ in acetone, and near 0 μS·cm^−1^ in toluene, which could well explain the weak electric response to ethanol and null to acetone and toluene.

## Conclusion

In summary, we have designed and prepared 2 novel nanocluster-based polymers that are composed of Ag nanoclusters linked with each other. Their crystal structures are determined by x-ray diffraction, which indicates that the coordination modes of ligands have an important effect on the self-assembly of cluster-based materials. Furthermore, the conductivity of the 2 polymers in the solid state could be altered by organic solvents. Interestingly, the Ag-CBPs exhibit a high electric response to methanol, suggesting that the cluster-based materials can be used to design sensitive sensors to detect organic solvents, especially methanol as demonstrated in this work. Finally, we have discussed the mechanistic insight into how the cluster-based materials work as a sensor in detecting organic solvents in the presence of ambient atmosphere. The synthesis and application of cluster-based materials enriches the application of metal nanoclusters.

## Data Availability

All data are available in the main text and the Supplementary Materials. Other relevant data are available from corresponding authors upon reasonable request.

## Supplementary Materials

Supplementary Materials Experimental Procedures. Scheme S1. The protecting organic ligands used in this work. Fig. S1. The connection between monomers in Ag_22_-CBP chains. Fig. S2. The coordination configurations of L1 ligand between the Ag_22_-CBP chains. Fig. S3. The coordination configurations of C≡C group in Ag_22_-CBP. Fig. S4. The coordination model of methanol in Ag_22_-CBP. Fig. S5. The minimum asymmetry structure in Ag_14_-CBP. Fig. S6. (A) The coordination model of L2 ligand in Ag_14_-CBP. Fig. S7. The Ag_12_ unit in Ag_14_-CBP monomer. Fig. S8. The molecule cavity filled with water molecules and hydrogen bonds in Ag_14_-CBP. Fig. S9. PXRD spectroscopy of Ag22-CBP. Fig. S10. PXRD spectroscopy of Ag22-CBP. Fig. S11. Optical photographs of Ag-CBP sensor in (A) flat and (B) bending state. Fig. S12. Schematic diagram and scheme of real-time sensing setup. Fig. S13. The dynamic response over Ag_16_-CBP thin film for mixtures of methanol and water. Fig. S14. The dynamic response and recovery curves of Ag_22_-CBP thin film for different organic solvents: (A) ethanol, (B) methanol, (C) acetone, (D) toluene, and (E) ultrapure water (resistance of 18.2 MΩ·cm), measured at 0.1-V bias. Fig. S15. The dynamic response and recovery curves over Ag_16_-CBP thin film for organic solvents: (A) ethanol and (B) methanol, measured under 0.1-V bias.
