# Supplementary material for: Self-Assembly of Silver Clusters into One- and Two-Dimensional Structures and Highly Selective Methanol Sensing
Source: Research (Wash D C). 2022 Dec 21;2022:0018. doi: 10.34133/research.0018 (PMC11407582; doi:10.34133/research.0018)
Supplement: Supplementary Materials — Experimental Procedures. Scheme S1. The protecting organic ligands used in this work. Fig. S1. The connection between monomers in Ag22-CBP chains. Fig. S2. The coordination configurations of L1 ligand between the Ag22-CBP chains. Fig. S3. The coordination configurations of C≡C group in Ag22-CBP. Fig. S4. The coordination model of methanol in Ag22-CBP. Fig. S5. The minimum asymmetry structure in Ag14-CBP. Fig. S6. (A) The coordination model of L2 ligand in Ag14-CBP. Fig. S7. The Ag12 unit in Ag14-CBP monomer. Fig. S8. The molecule cavity filled with water molecules and hydrogen bonds in Ag14-CBP. Fig. S9. PXRD spectroscopy of Ag22-CBP. Fig. S10. PXRD spectroscopy of Ag22-CBP. Fig. S11. Optical photographs of Ag-CBP sensor in (A) flat and (B) bending state. Fig. S12. Schematic diagram and scheme of real-time sensing setup. Fig. S13. The dynamic response over Ag16-CBP thin film for mixtures of methanol and water. Fig. S14. The dynamic response and recovery curves of Ag22-CBP thin film for different organic solvents: (A) ethanol, (B) methanol, (C) acetone, (D) toluene, and (E) ultrapure water (resistance of 18.2 MΩ·cm), measured at 0.1-V bias. Fig. S15. The dynamic response and recovery curves over Ag16-CBP thin film for organic solvents: (A) ethanol and (B) methanol, measured under 0.1-V bias. [file research.0018.f1.pdf]

Supplementary Information

Self-Assembly of Silver Clusters into One- and Two-Dimensional Structures and Highly Selective Methanol Sensing

Zhaoxian Qin,[1,4#] Zhiwen Li,[1,4#] Sachil Sharma,[1] Yongwu Peng,[3] Rongchao Jin[2]* and Gao Li[1,4,]**

1 State Key Laboratory of Catalysis, Dalian Institute of Chemical Physics, Chinese Academy of Sciences, Dalian 116023, China

2 Department of Chemistry, Carnegie Mellon University, Pittsburgh, PA 15213, USA

3 College of Materials Science and Engineering and College of Chemical Engineering, Zhejiang University of Technology, Hangzhou 310014, China

4 University of Chinese Academy of Sciences, 100049 Beijing, China.

#Z, Qin and Z. Li contribute to this work equally.

*Correspondence: gaoli@dicp.ac.cn (G. Li), rongchao@andrew.cmu.edu (R. Jin)

Table of Contents for the Supplementary Information

Experimental Procedures 1

Materials 1

Synthesis of 3-(prop-2-yn-1-yloxy)benzonitrile (abbreviated as H-L1)................1

Synthesis of Ag-L1 precursor 1

Synthesis of Ag-L2 precursor 2

Synthesis of Ag_{22}-CBP and Ag_{16}-CBP. 2

Sensor fabrication. 2

X-ray Crystallographic Analysis...2

Supplementary Figures 4

Experimental Procedures

Materials

All chemicals were commercially available and were used as received without further purification. 3-hydroxybenzonitrile (99%), 2-hydroxybenzonitrile (99%), Silver trifluoroacetate ($AgCO_2CF_3$, 99%), Silver tetrafluoroborate ($AgBF_6$, 99%), 1-(3-mercaptoprop-1-en-2-yl)-2-methoxypyridin-1-ium bromide (99%), methanol (HPLC grade, 99.9%), ethanol (HPLC grade, 99.9%), acetone (HPLC grade, 99.9%) and toluene (HPLC grade, 99.9%) were purchased from Adamas-beta®. Hydroxypropyl methylcellulose (HPMC) was received from Aladdin. HL1, L1-Ag and L2-Ag were synthesized according to reported methods[1]. Polyethylene terephthalate (PET, thickness of 125 μm) was obtained from Kangde Xin Composite Material Group. Ultrapure water (resistance 18.2 MΩ•cm) was purified with a Barnstead Nanopure Di-water TM system.

Synthesis of 3-(prop-2-yn-1-yloxy)benzonitrile (abbreviated as H-L1)

Generally, 2-hydroxybenzonitrile (1.19 g) was refluxed with K_2CO_3 (2.07 g) and 3-bromoprop-1-yne (1.475 g) in acetone for 24 h. Then, the solid was removed by filtration, and the filtrate was dried by rotary evaporation. The crude product was further purified through silica gel with dichloromethane as eluent. ^{1}H NMR (δ, CD_3OD): 2.87 (1H, H-C≡C), 4.25 (2H, $-CH_2-$), 7.79-8.71 (4 H, H-Ar).

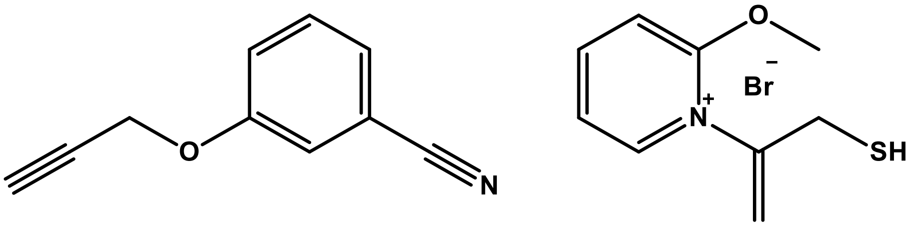

H-L1 H-L2

Scheme S1. The protecting organic ligands used in this work.

Synthesis of Ag-L1 precursor

Typically, H-L1 was dissolved with NEt_3 (1 eq.) in dichloromethane followed by the dropwise addition of $AgBF_4$ (1 eq. in methanol) under stirring. The mixture was kept stirring in dark for an hour. Subsequently, a white solid was obtained by rotary evaporation. The neat product was obtained by

washing the white crude mixture with methanol and ethyl ether.

Synthesis of Ag-L2 precursor

1-(3-mercaptoprop-1-en-2-yl)-2-methoxypyridin-1-ium bromide (abbreviated as H-L2, 23 mg, 0.1 mmol) ligand was dissolved into 10 mL MeOH at first and followed by the addition of $AgBF_4$ (19.4 mg, 0.1 mmol) dispersed into 10 mL MeOH dropwise, giving a light-yellow suspension. A few minutes later, the precipitate was in reactor was removed by filtration. And to the clear filtrate ~1 eq. NEt_3 (10 mg, 0.1 mmol) and $AgBF_4$ (38.8 mg, 0.2 mmol) were added subsequently and slowly. The precursor noted as Ag-L2 was obtained as a gray solid precipitate, which was separated and washed with MeOH and ethyl ether subsequently.

Synthesis of Ag_{22}-CBP and Ag_{16}-CBP.

All the operations were carried out in dark. Generally, 10 mg of Ag-L1 was dispersed into 5 mL of MeOH, followed by the addition of $AgCO_2CF_3$ (88 mg, 0.4 mmol, dissolved in 5 mL MeOH). A white suspension was generated. After 20 min later, filtration of the mixture gave a light-yellow solution, which was then exposed to ethyl ether for crystallization in dark in a refrigerator. Transparent yellow block-like crystals of $\{Ag_{22}(L1)_8(CO_2CF_3)_{14}(CH_3OH)_2\}_n$ (denoted as Ag_{22}-CBP) were obtained in a few weeks.

Ag_{16}-CBP was obtained by a similar procedure. Briefly, 10 mg Ag-L2 (dispersed in 5 mL MeOH) was used in the preparation of Ag_{16}-CBP. Light-yellow plate-like crystals of $\{[Ag_{12}(L2)_2(CO_2CF_3)_{14}(H_2O)_4(AgCO_2CF_3)_4](HNEt_3)_2\}_n$ (denoted as Ag_{16}-CBP) were obtained after weeks in dark in a refrigerator. The synthetic yields of Ag_{22}-CBP and Ag_{16}-CBP were 41% and 62% (based on consumption of $AgCO_2CF_3$), respectively.

Sensor fabrication.

For the preparation of the cluster thin-film sensor, 30 mg of the clusters was first dispersed in 5 mL of ethanol and then, 5 mL of hydroxyl propyl methyl cellulose (HPMC) aqueous solution (4 mg mL^{-1}) was added to adjust the viscosity. Next, 3 mL of cluster dispersion obtained from the previous step was dripped on polyethylene terephthalate (PET) and dried at 60 ºC in an oven for 10 min. Finally, Cu wires were attached to the two ends of the film for connecting to the power supply in electrical

measurements. The film of Ag-CBP sensors was sprayed into solvents. The pure solvents of methanol, ethanol, acetone and toluene in HPLC grade and ultrapure water (resistance 18.2 MΩ•cm) were used for the dynamic response and recovery of Ag-CBP sensors. The response time was 8 s, and the recovery time was 14 s. The sensitivity could be calculated as relative capacitance change for $\delta=\frac{|I_x-I_0|}{I_0}\times 100$, where δ is relative current change, I_x and I_0 are measured currents when the sensor contacted with solvents and the initial current of free sensor (before dipping into solvents), respectively.

The conductivity of the samples in solution was determined by a DDS-307 conductivity meter. Crystal samples were dispersed into different solution and then filtrated with a pinhole membrane filter, generating saturated solutions to be tested. Before the measurement, the conductivity meter was calibrated by a standard solution of 1408 μS cm^{-1}.

X-ray Crystallographic Analysis

The crystal diffraction data was collected on an Xcalibur, Atlas, Gemini ultra diffractometer. Data reduction, cell refinement and experimental absorption correction were performed with the software package of CrysAlis^{pro} (1.171.39.38a). The structures were solved by intrinsic phasing methods by ShelXT 2015[2] and refined against F^2 by full-matrix least-squares by ShelXL 2015[3]. All non-hydrogen atoms were refined anisotropically. Hydrogen atoms were generated geometrically. All the calculations were carried out using the program package of Olex2 (*ver.* 1.2.10)[4].

The crystal data for [$C_{55}H_{28}Ag_{11}F_{21}N_4O_{19}$], monoclinic, *C2/c*, a = 31.2047 Å, b = 14.4204 Å, c = 30.1096 Å, β = 101.376 °, V = 13282.7 $Å^3$, Z = 8, T = 140 K, 29494 reflections measured, R_1 = 0.0543, wR_2 = 0.1452.

The crystal data for [$C_{33}H_{18}Ag_8F_{27}N_2O_{21}S$], triclinic, *P-1*, a = 12.5661 Å, b = 15.2008 Å, c = 17.6631 Å, α = 84.290(3) °, β = 83.029(4) °, γ = 67.249(4) °, V = 3083.3(2) $Å^3$, Z = 2, T = 293 K, 42762 reflections measured, R_1 = 0.0678, wR_2 = 0.1786.

Supplementary Figures

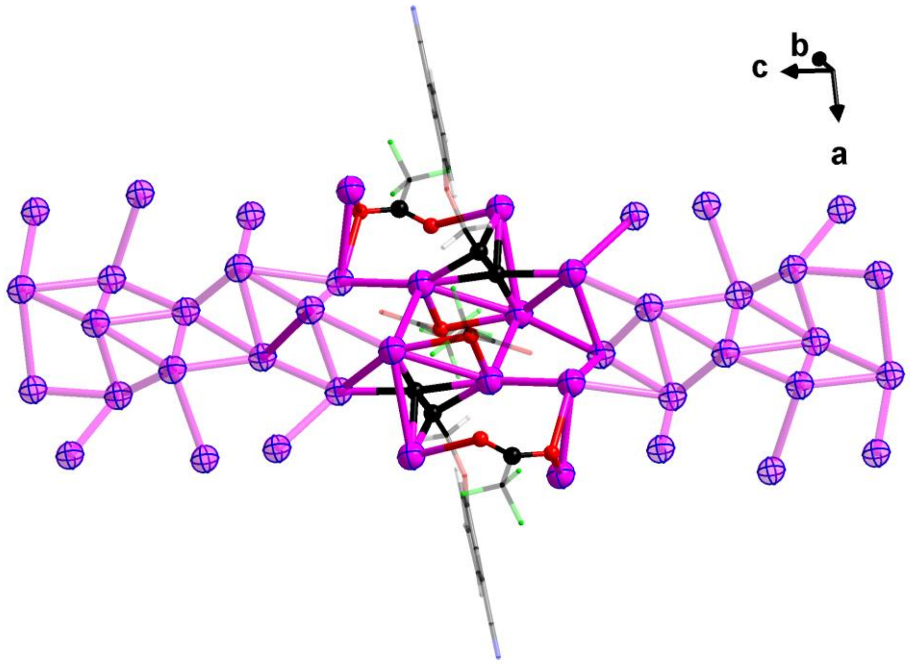

Figure S1. The connection between monomers in Ag_{22}-CBP chains.

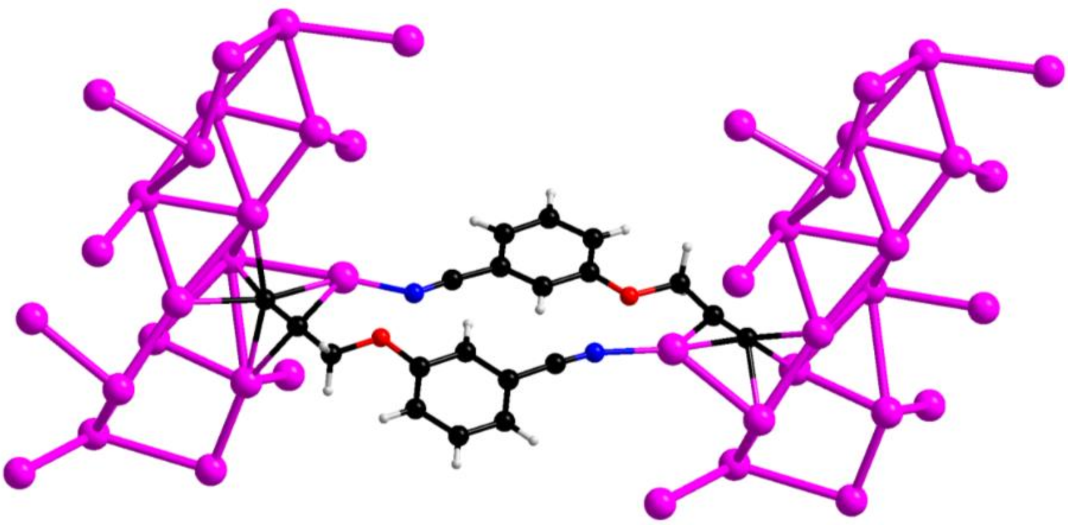

Figure S2. The coordination configurations of L1 ligand between the Ag_{22}-CBP chains. Color code: Ag, pink; C, black; O, red; N, blue; H, white.

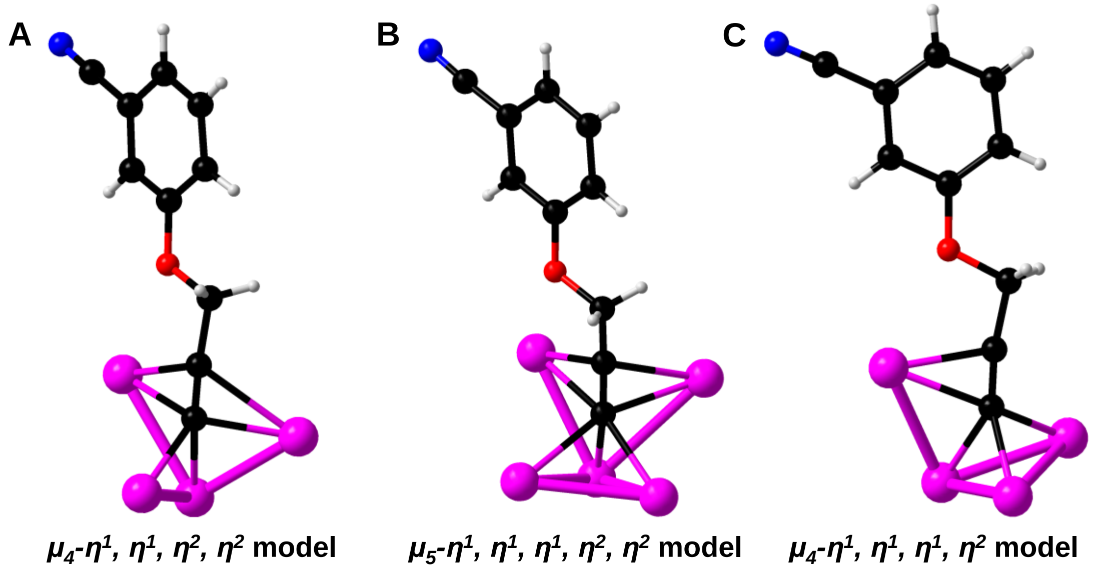

Figure S3. The coordination configurations of C≡C group in Ag_{22}-CBP. Color code: Ag, pink; C, black; O, red; N, blue; H, white.

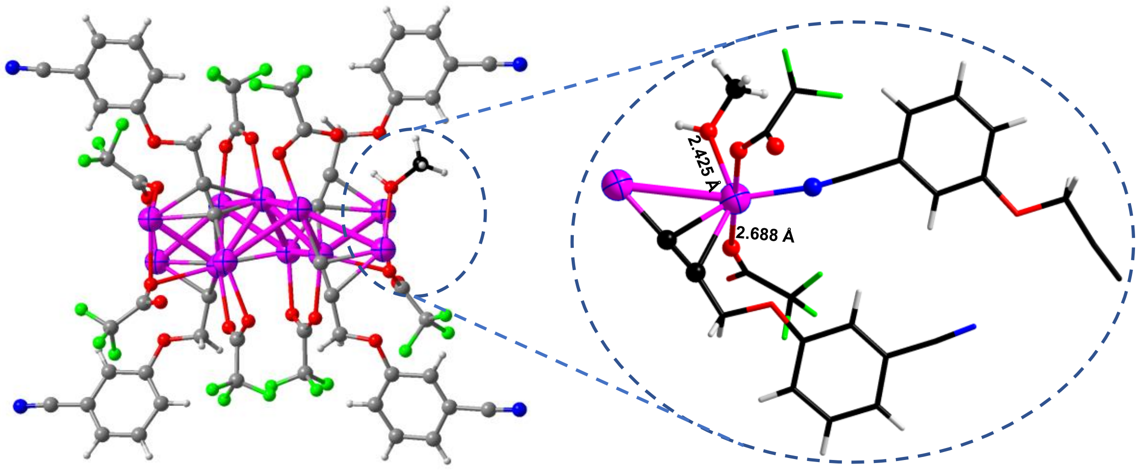

Figure S4. The coordination model of methanol in Ag_{22}-CBP.

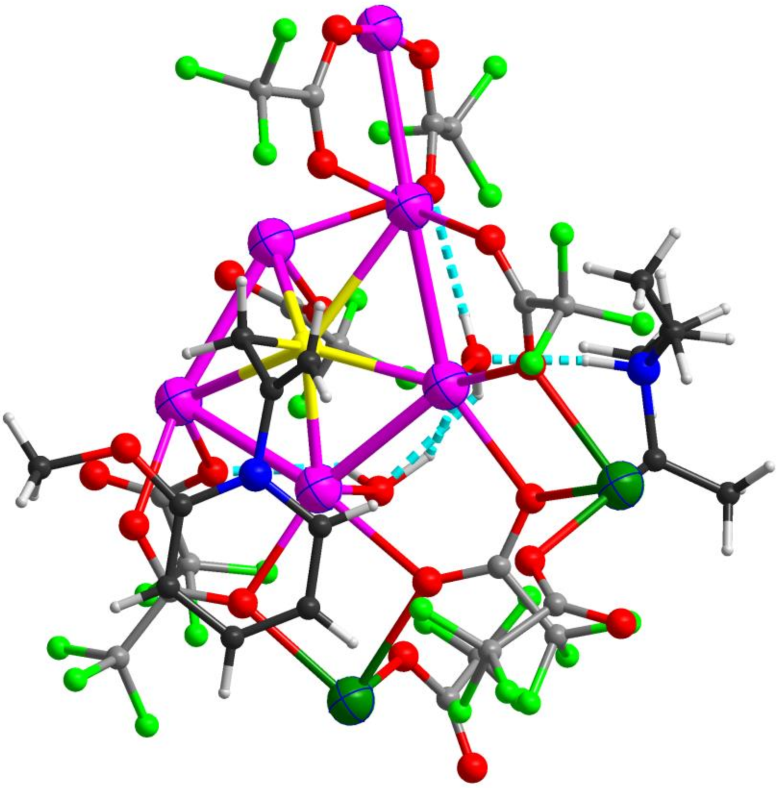

Figure S5. The minimum asymmetry structure in Ag_{14}-CBP. Color code: Ag, pink and dark green; C, black and gray; O, red; S, yellow; N, blue; F, light green; H, white.

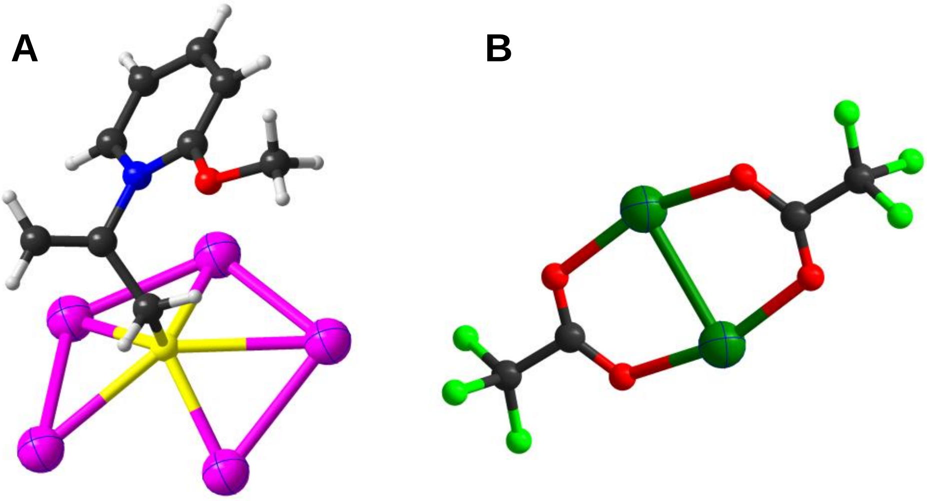

Figure S6. (A) The coordination model of L2 ligand in Ag_{14}-CBP. (B) The Ag_2 unit. Color code: Ag, pink and dark green; C, black; O, red; S, yellow; N, blue; F, light green; H, white.

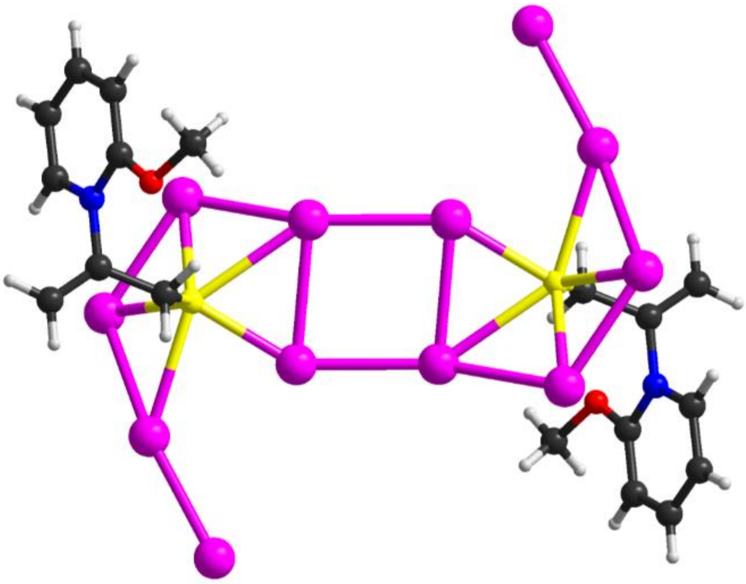

Figure S7. The Ag_{12} unit in Ag_{14}-CBP monomer. Color code: Ag, pink; C, black; O, red; S, yellow; N, blue; H, white.

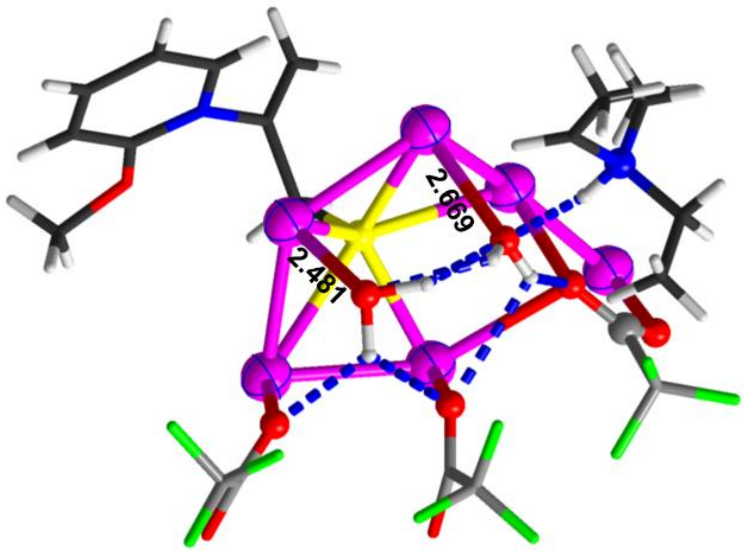

Figure S8. The molecule cavity filled with water molecules and hydrogen bonds in Ag_{14}-CBP.

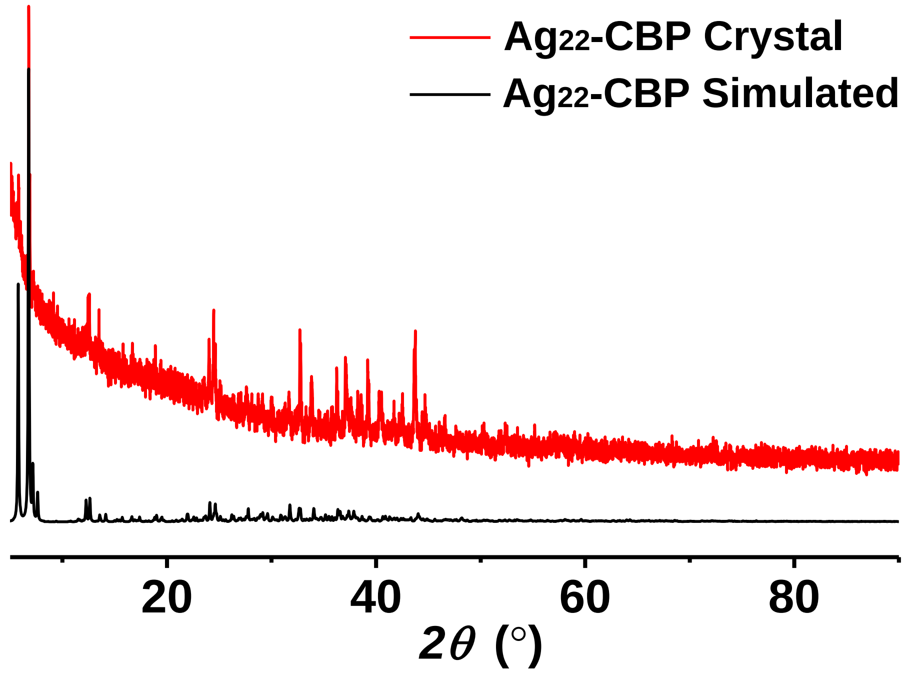

Figure S9. Powder X-ray diffraction (PXRD) spectroscopy of Ag22-CBP. Red curve: tested with crystals. Black curve: simulated according to single crystal structure of Ag22-CBP.

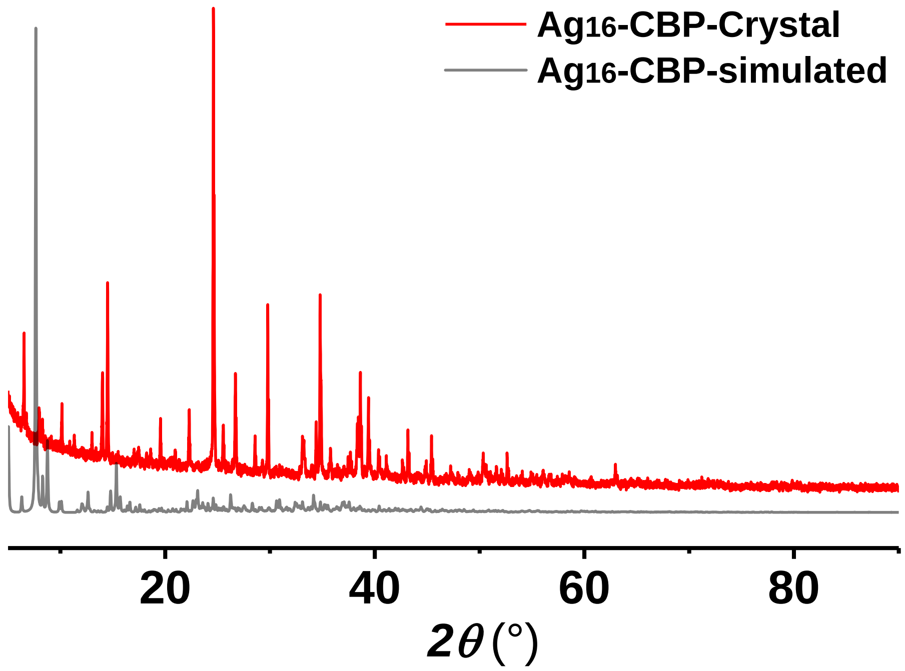

Figure S10. Powder X-ray diffraction (PXRD) spectroscopy of Ag22-CBP. Red curve: tested with crystals. Black curve: simulated according to single crystal structure of Ag22-CBP.

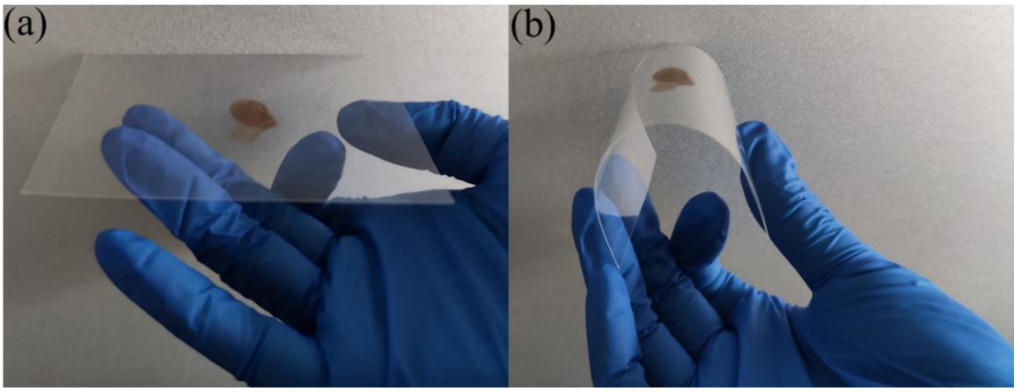

Figure S11. Optical photographs of Ag-CBP sensor in (a) flat and (b) bending state.

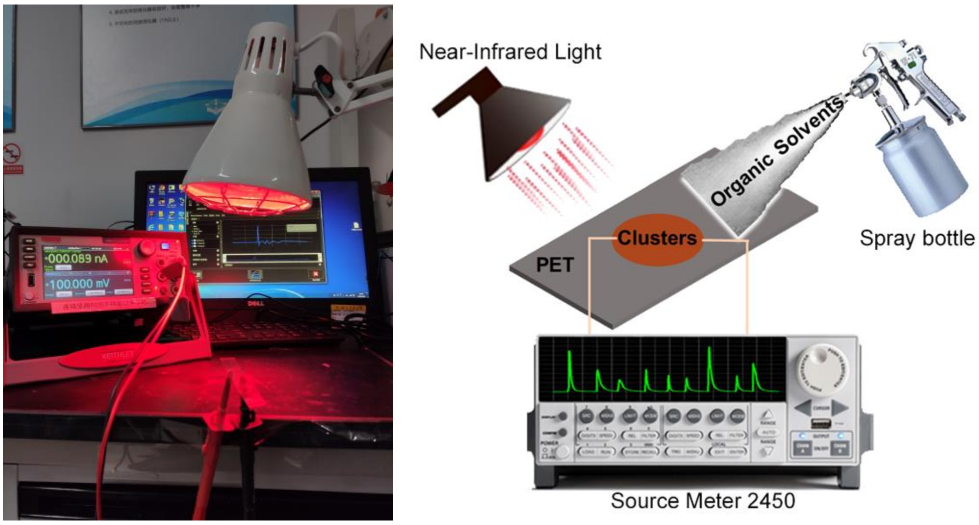

Figure S12. Schematic diagram and scheme of real-time sensing setup. Near-Infrared light was used to speed the remove of solvents only after test.

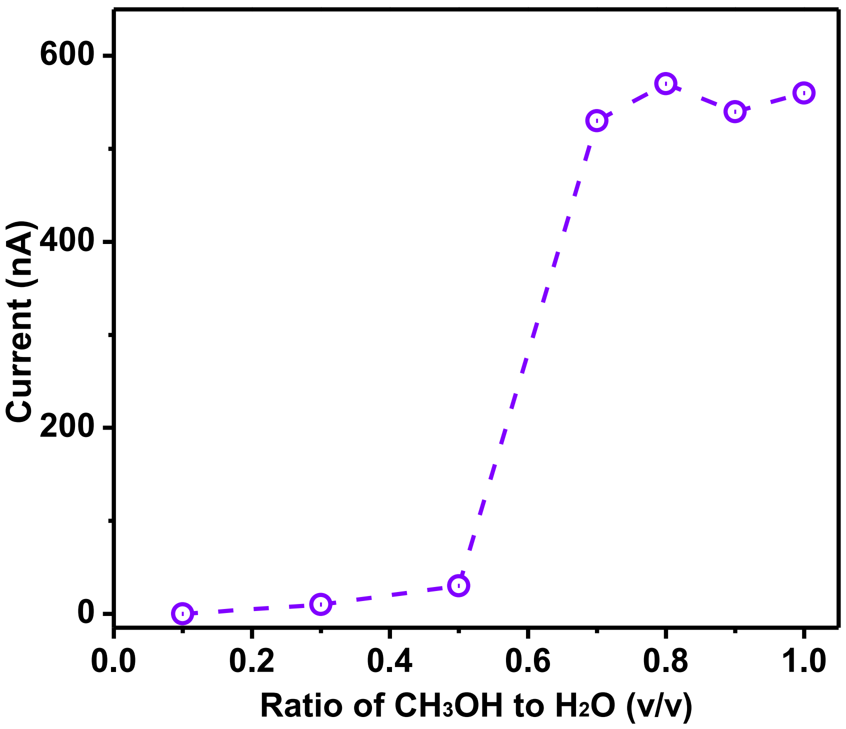

Figure S13. The dynamic response over Ag_{16}-CBP thin film for mixtures of methanol and water.

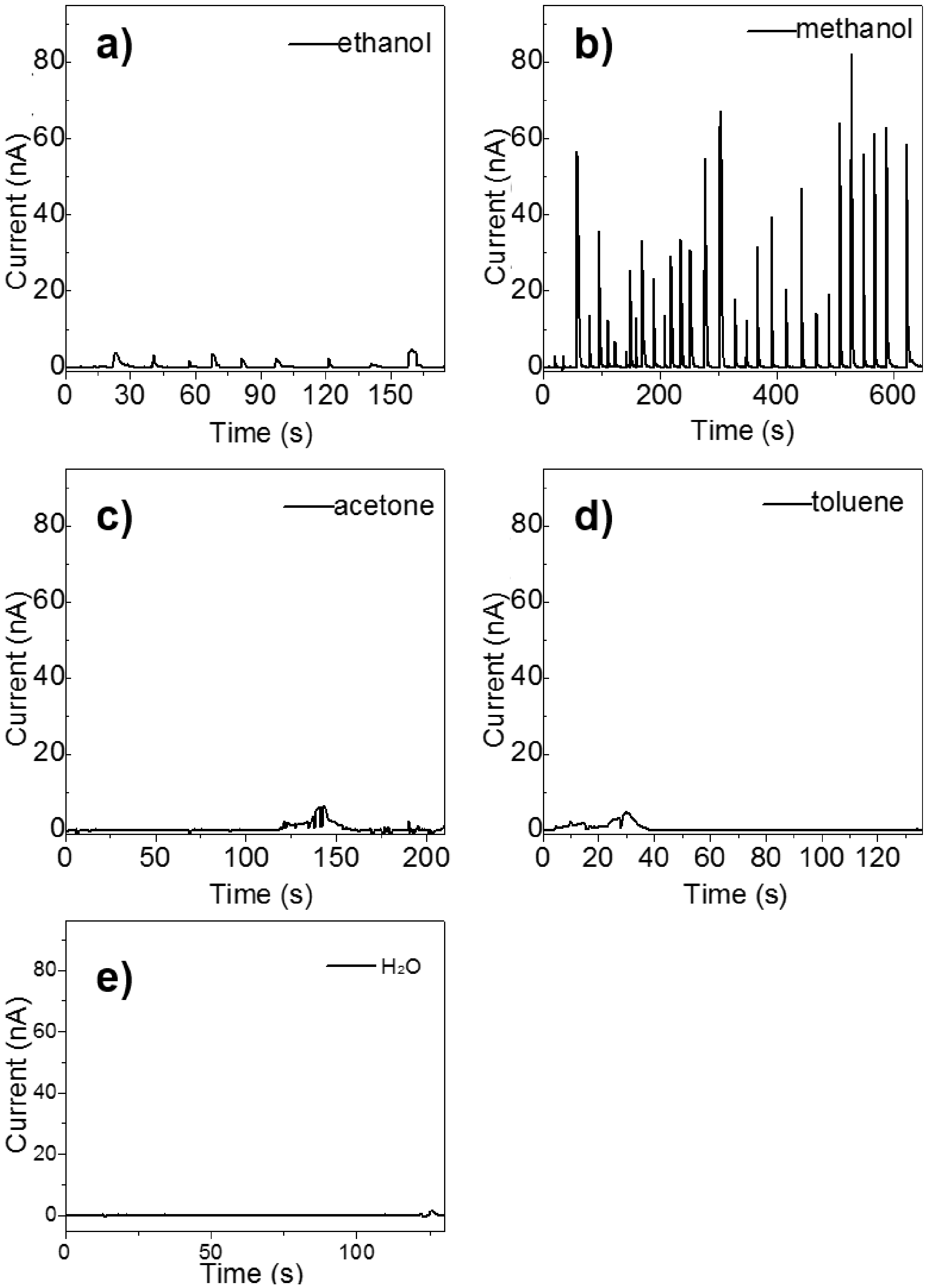

Figure S14. The dynamic response and recovery curves of Ag_{22}-CBP thin film for different organic solvents: (a) ethanol, (b) methanol, (c) acetone, (d) toluene, and (e) ultrapure water (resistance 18.2 MΩ•cm), measured at 0.1 V bias.

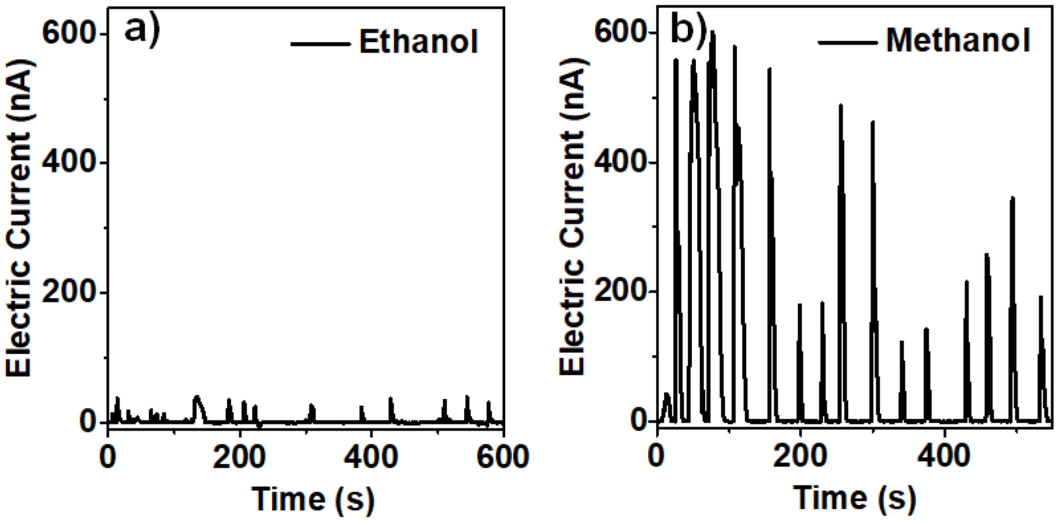

Figure S15. The dynamic response and recovery curves over Ag_{16}-CBP thin film for organic solvents: (a) ethanol and (b) methanol, measured under 0.1 V bias.

References

1. Qin, Z., Sharma, S., Wan, C.Q., Malola, S., Xu, W.W., Hakkinen, H., Li, G. *Angew. Chem. Int. Ed.* **60**, 970 (2021).

2. Sheldrick, G.M., *Acta Crystallogr. A Found. Adv.* **71**, 3 (2015).

3. Sheldrick, G.M., *Acta Crystallogr. C Struct. Chem.* **71**, 3 (2015).

4. Dolomanov, O.V., Bourhis, L.J., Gildea, R.J., Howard, J.A.K., Puschmann, H., *J. Appl. Crystallogr.* **42**, 339 (2009).
